# Genital Herpes in an HIV-Positive Patient Mimicking Donovanosis: A Case Report

**DOI:** 10.7759/cureus.115678

**Published:** 2026-09-02

**Authors:** Julia Falchetti da Silva, Nicolle Kayse Ferreira Araujo, Pamela Bacellar Rosenblat, Sandra D Teixeira Araujo Moraes

**Affiliations:** 1 Obstetrics and Gynecology, Hospital e Maternidade Amador Aguiar, Osasco, BRA

**Keywords:** donovanosis, genital herpes, genital ulcers, granuloma inguinale, hiv diseases, vulvar lesion

## Abstract

Genital herpes is one of the most common sexually transmitted infections worldwide, primarily driven by herpes simplex virus type 2 (HSV-2). In immunocompetent individuals, the disease typically manifests as self-limiting vesicular lesions that progress to painful ulcerations. However, in patients living with human immunodeficiency virus (HIV), the clinical presentation can be remarkably atypical, chronic, and severe due to impaired cell-mediated immunity. In this specific population, hypertrophic or extensive vegetative ulcerations frequently mimic other infectious diseases and malignancy, creating a complex diagnostic dilemma for clinicians. This case report describes a 41-year-old woman who presented with persistent, chronic ulcerated vulvar lesions. Due to the aggressive and granulomatous clinical appearance of the lesions, the initial differential diagnoses focused heavily on donovanosis (granuloma inguinale) and vulvar malignancy. To establish an accurate diagnosis, an extensive clinical investigation was performed. Polymerase chain reaction (PCR) testing definitively confirmed HSV-2 infection. Upon initial evaluation, the patient was found to have a newly diagnosed HIV infection during the current admission; however, she later disclosed a prior diagnosis of HIV dating back to 2005, which she had not previously reported. The patient was managed with targeted intravenous acyclovir therapy, which resulted in significant and complete clinical improvement of the vulvar lesions. This case emphasizes the high variability of opportunist-associated cutaneous presentations in the context of advanced immunosuppression. It underscores the vital role of molecular diagnostics and tissue biopsies in distinguishing atypical herpes simplex virus presentations from other sexually transmitted infections and malignancies, ensuring timely antiviral intervention.

## Introduction

Genital herpes is one of the most common sexually transmitted infections (STIs) worldwide [1-3]. Herpes simplex virus type 2 (HSV-2), an enveloped DNA virus, is the predominant cause of the disease and it is primarily transmitted through direct contact [1,2]. The classical clinical features consist of ulcerative lesions, and the typical symptoms include burning pain and pruritus [1,3,4]. A genital ulcer is clinically defined as a mucosal or cutaneous breach affecting the genital tract [4].

HSV-2 has a well-documented biological and epidemiological association with HIV infection. In co-infected individuals, impaired immunologic control and low CD4+ T-cell counts characteristically trigger more frequent and severe viral shedding and reactivation [2,4]. Atypical and chronic genital ulcers may occur, particularly in immunocompromised patients, making clinical diagnosis challenging [2,5,6]. There are numerous potential differential diagnoses for genital herpes; while malignancy is one possibility, other infectious diseases such as syphilis, chancroid, lymphogranuloma venereum, and granuloma inguinale must also be considered [4,5]. Donovanosis, also known as granuloma inguinale, is an STI characterized by chronic genital ulcerations with granulomatous infiltration, and may be included in the differential diagnosis of persistent ulcerative genital lesions, particularly in endemic or epidemiologically relevant settings [4,6,7].

Given the global prevalence of genital ulcers, which present with a wide range of differential diagnoses and clinical manifestations, we report a case of an HIV-positive woman with chronic ulcerated vulvar lesions initially raising suspicion for donovanosis and vulvar malignancy, in whom the final diagnosis was genital herpes, highlighting the diagnostic challenges associated with atypical genital herpes in the setting of HIV infection.

## Case presentation

A 41-year-old White woman from Osasco, São Paulo, presented to the Hospital and Maternity Amador Aguiar (HMAA) on November 18, 2021, with vulvar lesions persistent for two months with intense local pruritus. She denied any prior treatment and reported no comorbidities.

**Figure 1:**
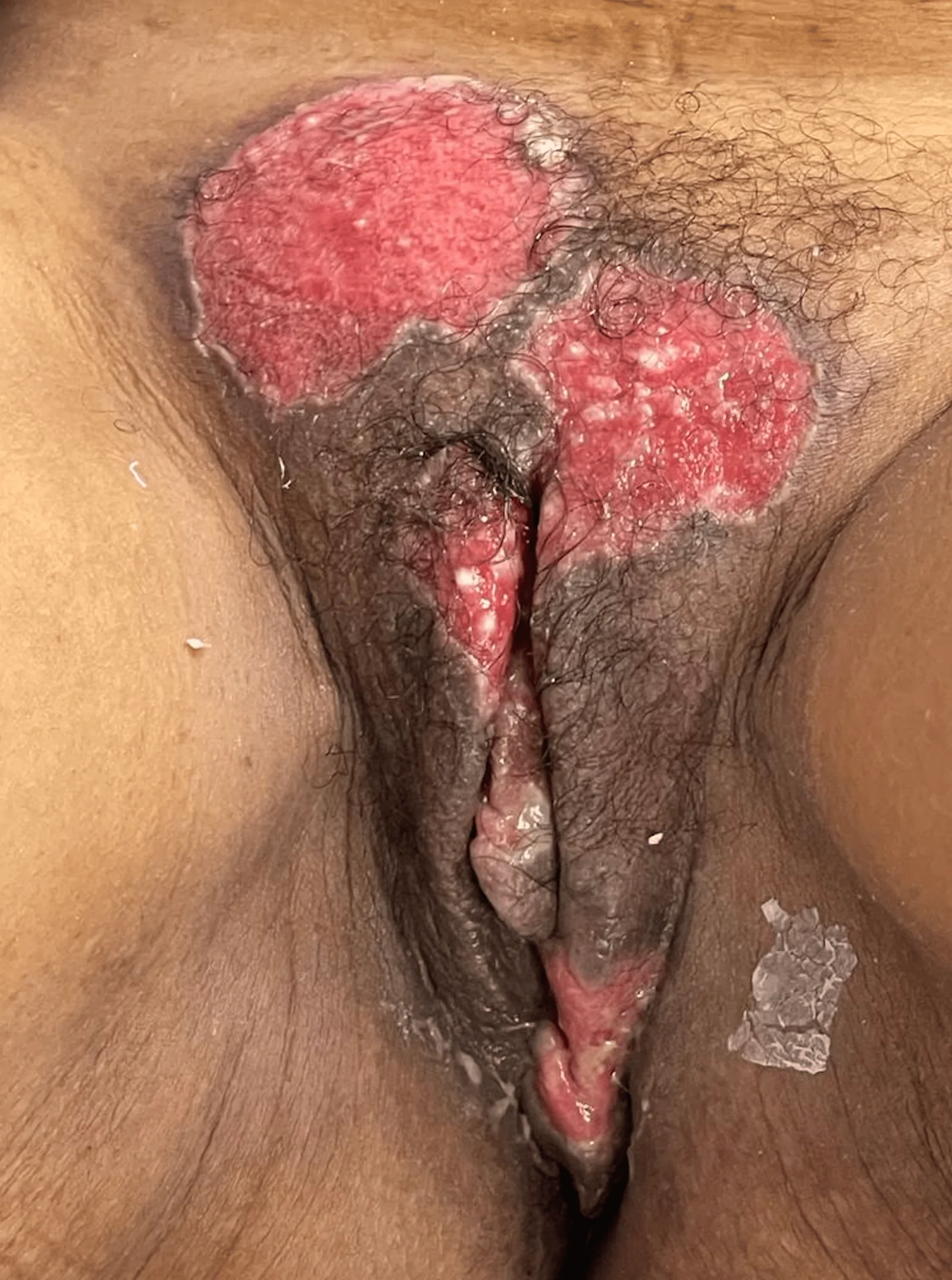
Genital herpes in a female patient Genital lesion presentation upon hospital admission, with a reported duration of two months.

Physical examination revealed multiple large, confluent, erythematous, ulcerative lesions involving the vulvar region, with the largest measuring approximately 5-6 cm in diameter. The lesions were predominantly superficial, with areas of deeper ulceration (Figure 1). The ulcer base was bright erythematous, denuded, and moist, with focal areas of whitish exudate. The borders were irregular but relatively well-demarcated, without obvious rolled or indurated edges. Initially, the differential diagnoses included vulvar cancer, genital herpes and donovanosis.

On Day 1, a lesion culture was performed, and antibiotic therapy was initiated as empirical syndromic management for suspected concurrent STIs, utilizing intravenous ceftriaxone 1 g daily, oral doxycycline 100 mg every 12 hours, and a single 1 g dose of oral azithromycin.

On Day 2, following the initiation of antimicrobial treatment, a biopsy was performed concurrently with rapid testing for syphilis and HIV. The rapid screening test for syphilis was negative, whereas the initial HIV screening was positive; a subsequent enzyme-linked immunosorbent assay (ELISA) was performed. On Day 5, the ELISA assay confirmed the positive result for HIV. Following this confirmation, the patient revealed a known history of HIV infection since 2005, diagnosed during a previous pregnancy. She admitted to withholding this information at the time of admission and denied any history of prior antiretroviral therapy (ART), stating that she had not accepted the diagnosis at that time.

Also on Day 5, the microbiological culture isolated Gram-positive cocci and Gram-negative bacilli, specifically identifying Pseudomonas putida susceptible to gentamicin and piperacillin/tazobactam. The lesion swab, submitted for polymerase chain reaction (PCR) testing, confirmed the presence of HSV-2. Concurrently, the vulvar biopsy was consistent with chronic ulcerated vulvitis with an acute exacerbation and did not reveal multinucleated giant cells. Additionally, a perianal biopsy demonstrated a low-grade squamous intraepithelial lesion, while a cervical biopsy revealed high-grade squamous intraepithelial neoplasia (CIN III) with glandular extension.

Following receipt of the test results and confirmation of normal renal function, therapy with intravenous piperacillin/tazobactam and acyclovir was initiated, whereas the previously administered antibiotics were suspended in light of the broad-spectrum and targeted coverage provided by the newly introduced regimen. Due to the unavailability of intravenous acyclovir at the maternity unit, as well as the inability to perform CD4 lymphocyte count and HIV-RNA viral load testing, the patient was referred to a specialized center for comprehensive HIV, vulvar lesion, and CIN III with glandular extension management. Clinical improvement of the vulvar lesions was observed following the administration of intravenous acyclovir in conjunction with the aforementioned antimicrobials. Furthermore, the patient continued to receive longitudinal follow-up at the specialized care center for the ongoing management of her remaining comorbidities, including ART for HIV infection.

## Discussion

Genital herpes caused by HSV-1 or HSV-2 ranks among the most prevalent STIs globally [1,3], with HSV-2 being the more common etiology [2,8]. Anatomical factors within the female genital tract result in a heightened risk of transmission from an infected partner compared to the risk faced by men [8]. The classic clinical presentation involves macular or papular lesions appearing days post-exposure that progressively evolve into vesicles and ulcers, persisting for up to three weeks [1,5]. Genital lesions can be exceptionally painful, frequently presenting with vulvar edema in women, burning sensations, and dysuria [5,8].

The diagnosis of genital herpes is largely clinical, relying on patient history and characteristic physical findings [5,8]. Nonetheless, many patients present with atypical manifestations that can easily mimic other genital diseases, whether infectious or non-infectious [8]. Laboratory diagnostics play a critical role in the definitive diagnosis of HSV infection; thus, viral swabs of genital lesions should be collected for PCR testing [1,2,5,8,9].

HIV and HSV infections are major global health concerns and lifelong infections that share common risk factors and frequently involve the genital tract [9,10]. Due to the established association between these diseases, patients with a newly diagnosed HSV infection should undergo an STI screening, including HIV [5,9]. Untreated individuals living with HIV may develop severe immunodeficiency and are at increased risk of more severe HSV manifestations [4,6,10].

In individuals with HIV infection, genital ulcerative lesions may have atypical morphology, prolonged duration, and increased severity, requiring careful consideration of alternative diagnoses [4,6,7], as illustrated by this case report, in which the ulcer mimicked a donovanosis infection. Caused by Klebsiella granulomatis, granuloma inguinale characteristically presents with ulcerovegetative lesions, most commonly involving the labia minora and vaginal fourchette in female patients [4,7].

## Conclusions

Atypical genital herpes in patients with HIV infection presents significant diagnostic challenges, particularly when chronic, extensive, or hypertrophic ulcerative lesions mimic other infectious or malignant vulvar conditions. Because diagnostic delays frequently stem from patient nondisclosure or unrecognized immunosuppression, universal baseline HIV and other STI screening are indicated for non-healing genital lesions, regardless of initial patient reporting. In these complex cases, HSV-2 must remain a primary suspect. While PCR testing of lesion swabs provides a superior diagnostic pearl over visual inspection, a concurrent diagnostic tissue biopsy is critical. Histopathology is essential to exclude or identify malignancy, which is aggressively exacerbated by HIV-mediated mucosal immune dysfunction. Early clinical recognition must translate into aggressive management realities, including the use of intravenous antiviral therapy for severe or hypertrophic presentations, targeted treatment for secondary bacterial superinfections (such as Pseudomonas putida), and ART to restore cellular immunity. Ultimately, optimizing long-term patient recovery requires a comprehensive, multidisciplinary approach that bridges gynecologic and infectious disease, ensuring robust long-term suppressive antiviral therapy and strict longitudinal follow-up to support complete re-epithelialization and prevent recurrence.
